# The Fabrication of Micro Beam from Photopolymer by Digital Light Processing 3D Printing Technology

**DOI:** 10.3390/mi11050518

**Published:** 2020-05-20

**Authors:** Ishak Ertugrul

**Affiliations:** Department of Mechatronics, Mus Alparslan University, 49250 Mus, Turkey; i.ertugrul@alparslan.edu.tr

**Keywords:** digital light processing, projection micro-stereolithography, 3D printing, micro beam, fabrication

## Abstract

3D printing has lately received considerable critical attention for the fast fabrication of 3D structures to be utilized in various industrial applications. This study aimed to fabricate a micro beam with digital light processing (DLP) based 3D printing technology. Compound technology and essential coefficients of the 3D printing operation were applied. To observe the success of the DLP method, it was compared with another fabrication method, called projection micro-stereolithography (PμSL). Evaluation experiments showed that the 3D printer could print materials with smaller than 86.7 µm dimension properties. The micro beam that moves in one direction (*y*-axis) was designed using the determined criteria. Though the same design was used for the DLP and PμSL methods, the supporting structures were not manufactured with PμSL. The micro beam was fabricated by removing the supports from the original design in PμSL. Though 3 μm diameter supports could be produced with the DLP, it was not possible to fabricate them with PμSL. Besides, DLP was found to be better than PμSL for the fabrication of complex, non-symmetric support structures. The presented results in this study demonstrate the efficiency of 3D printing technology and the simplicity of manufacturing a micro beam using the DLP method with speed and high sensitivity.

## 1. Introduction

The micro-electro-mechanical system (MEMS) is a process technology comprising of miniaturized mechanical and electronic parts, that includes the transformation of a measured mechanic signal into a readable signal. This signal may be force, pressure, heat, or chemical. MEMS has created serious innovations in micro- and nano-study fields since the early 1980s [1]. Primarily, MEMS has been improved for different implementations for example force, navigation, optical transmitting, radio frequency, biological and medical, microfluidics, and gyroscope applications [2,3,4,5,6]. Currently, MEMS has become an essential part of various study fields such as material, mechanical, and electrical engineering studies [7,8,9]. The MEMS device is usually formed of four components, micro-structures, micro-sensors, micro-actuators, and micro-electronics for data utilization [10,11].

MEMS technologies have been manufactured by traditional methods such as lithography, galvano forming, and photolithography etc. These methods are dependent on additive or subtractive procedures that operate minuscule capacities of materials in the shape of thin layers on the surface of silicon wafers [12,13,14,15,16]. These conventional techniques are extremely precise and appropriate for the production of planar geometries [17]. Although precise, these operations are associated with some drawbacks such as multiple processing steps, the requirement of a cleanroom, an advanced work environment, lengthy fabrication, inconsistency of flexible materials, and a costly fabrication process [18]. With the improvement of 3D printing-additive manufacturing, the fabrication costs and processing steps of MEMS devices have been gradually reduced. According to these improvements, it is possible to fabricate MEMS devices in atmospheric air without the need for multiple operations and cleanrooms [19,20]. Due to such advantages, it is now possible to use 3D printing technology for micro beam fabrication. 

3D printing technology has become progressively popular in the fabrication of MEMS, since it can be employed to manufacture complicated structures clearly from digital files, for instance computer-assisted design drawings. This technology helps to improve the design and production and especially facilitates the production and repair of complex parts using printing through layer- by-layer deposition of the constituent materials [21,22,23]. With the progress of 3D printing techniques, interest in using 3D printing for building MEMS systems has grown remarkably in the areas of biomedical, electronics, wearable devices, soft robots, and automotive applications [24,25,26,27,28,29]. Unlike traditional manufacturing processes such as machining and punching, 3D printing does not entail on-site process control, cutting tools, coolers, or other additional resources. One of the important factors of 3D printing methods is its capability to make miniaturized complex structural geometries using easy steps that are not achievable by traditional manufacturing methods. Besides that, 3D printing methods offer many other characteristics, for example flexibility in geometrical designs, excellent feature size and shapes, and the ability to print functionally classified materials [30,31].

Micro beams are utilized as important components of different sensing and actuation systems such as sensors, gyroscopes, micro actuators, and resonators [32,33,34]. Their easy geometries make them very advantageous in terms of design, and microfabrication. In many applications, ranging from residual stress measurement mass flow sensors to biomedical or DNA analysis, the sensing mechanism is linked to the sensitivity of the micro beam to some applied stimulation [35,36,37]. Many studies have been conducted, especially in the field of DNA [38,39,40]. In a study, a multi-scale analytical model was created to define the relationship between the surface mechanical features of DNA self-assembled 2D films and the detection signals of DNA-micro beam systems [41]. Micro beams exactly predict the dynamic properties of the device, such as its natural frequencies and forced- vibration response. 

Fabrication methods are very significant in designing and researching micro beams. Until now, conventional MEMS fabrication methods have been used, such as photolithography and surface micromachining, etc. [42,43]. These methods are usually time-consuming, with high fabrication costs, and multi-step fabrication processing. Alternatively, the 3D printing or additive manufacturing method solves these problems by manufacturing the structure directly. In recent years, various 3D printing methods have been employed, for instance frontal polymerization (FP), projection micro- stereolithography (PμSL), laser micro sintering (LMS), selective laser melting (SLM), etc. [44,45,46,47]. Among the different 3D printing techniques, the digital light processing (DLP) technique using photocurable resins is appealing, since it can be used to manufacture a single layer of the 3D object through spatially-controlled solidification using a projector light [48]. This light produces benefits such as fast fabrication, high sensitivity, and surface quality. Besides, it is feasible to adapt the final features of the printed object by only altering the photocurable resin formulations [49]. In this way, it is feasible to reach a large diversity of systems for the fabrication of structures with excellent features and functions [50]. 

In this study, we present our development on utilization of DLP technology for fast and highly sensitive production of a micro beam with sub-millimeter scale properties. The DLP and PμSL methods were compared based on the fabrication results. With this study, a micro beam was fabricated for the first time using the 3D printing method. It is expected that this paper will contribute to the current literature in terms of manufacturing a micro device through the use and comparison of different techniques.

This study is arranged as follows. Section 2 describes the design of the micro beam. Section 3 explains the fabrication process, results, and discussion. Section 4 displays the result of the studies.

## 2. Materials and Methods 

### 2.1. Design Conditions

The possibility of coupling thermal, electrical, and structural characterization by fabrication of a micro beam is accomplished with a model. For characterization, the displacement of the micro beam is produced by passing a current through a beam; heat is produced by the current, and the rise in temperature causes a displacement through thermal expansion. The displacement of the micro beam is formed in these situations. 

**Figure 1:**
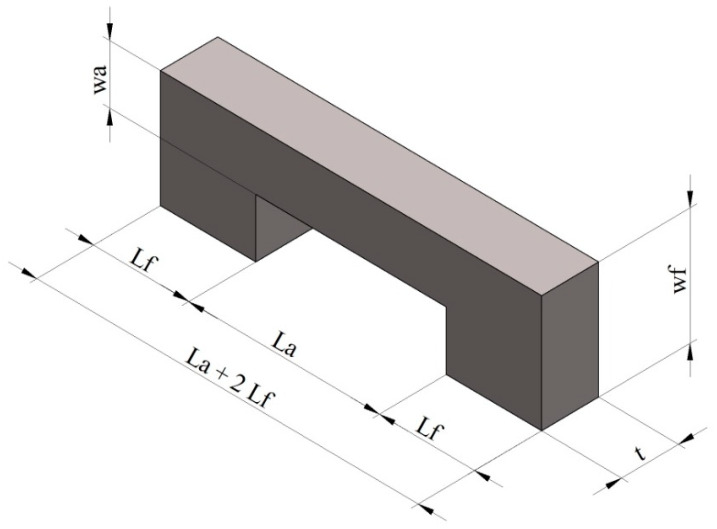
Dimensions of the micro beam.

The MEMS-based micro beam is designed to move in one direction (*y*-axis). For the beam to move on the *y*-axis, DC voltage must be applied. The feet of the micro beam at both ends are rigidly bound to a substrate, and DC voltage is applied at both ends. The applied voltage induces an electric current in the micro beam; current passing through the structure causes some retardation to the free flow of electrons by which energy is dissipated in the form of heat. This generated heat induces thermal stress on the beam and displaces the beam. The dimensioning and geometric structure of the micro beam, designed as a 3D (3-dimensional plane) using CAD software, is shown in Figure 1.

All the dimensions of our micro beam are shown in Table 1. These values were obtained with the measurements of the beam made with the DLP and PμSL methods. The same design was used for both methods. Nevertheless, as the supporting structures cannot be fabricated with the PμSL, the measurements concerning the support are shown in Section 3. Photopolymer materials were used for this study. These materials are frequently utilized in the field of MEMS because of their essential physical and electrical features.

### 2.2. Digital Light Processing Method

**Figure 2:**
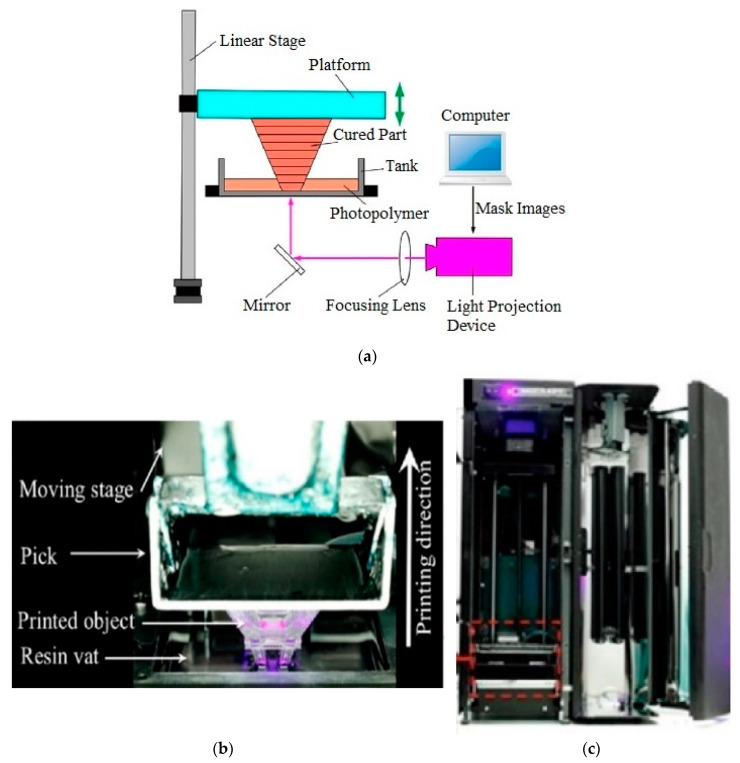
(**a**) Schematic of a digital light processing (DLP) system [53]; (**b**) 3D printing process in progress; (**c**) MiiCraft 3D printer.

Digital light processing (DLP) is a rapid additive fabrication technology with superior sensitivity. The processing basis of DLP technology is explained in [51,52] and can be briefly abstracted as follows. A commercial 3D printer, MiiCraft 125 (Rapid City, Canada), was utilized in this paper, as shown in Figure 2b.

As given in Figure 2a, the 3D pattern of the matter is first sliced into layers horizontally (in the *x*-axis). Thin layers are then transformed into 2D mask images. A light projection device is utilized to harden the photopolymer resin. This device employs a digital masking method to reflect a dynamically described mask image on the resin plane. With respect to [51], a bottom-up projection system has many benefits compared to a top-bottom system. In this system, the mask image is reflected on the bottom of a resin tank with cured resin at the bottom of the tank. This process continues until the desired design is created.

We established a bottom-up DLP system, as shown in Figure 2b. The DLP projector was equipped to provide a 400 nm full HD ultraviolet light source. The contrast ratio of the DLP system projector is 900:1. The XY resolution of the 3D printing device is 65 µm and the maximum building size 125 × 70 × 120 mm. With respect to [52,53], the masking method primarily occurs as three types: liquid crystal display (LCD), digital micro-mirror device (DMD), and liquid crystal on silicon (LcoS). In our improvement, the projector uses the DMD method. An optical reflector is employed to set the direction of the UV light. A position adjusting device is employed to set the position and behavior of the reflector. The adjustable angle range is ± 15°, and the accuracy is 0.003°. A flexible compressing device is used to press the resin vat. This device allows the vat to be lifted to a specific height. 

For the DLP method, IP-S resin, which is a photopolymer, was used as the material. This resin was designed to tend the double function of index-matching the dip fluid for final focusing of the object and photo-polymerizable, thus enabling the highest resolution at a given magnification. The elemental composition and fundamental features of the resin are given in Table 2 [54]. The composition was decided upon by using the procedure discussed in [55] and is significant for this study since it decided the x-ray absorption characteristics of the foam [56]. The foam structure extracted from the glass substrate was qualified using optical microscopy and scanning electron microscope (SEM). SEM sample preparation included a sputter coating of 30 nm thick gold to allow electrical charge conductivity while imaging.

### 2.3. PμSL Method

**Figure 3:**
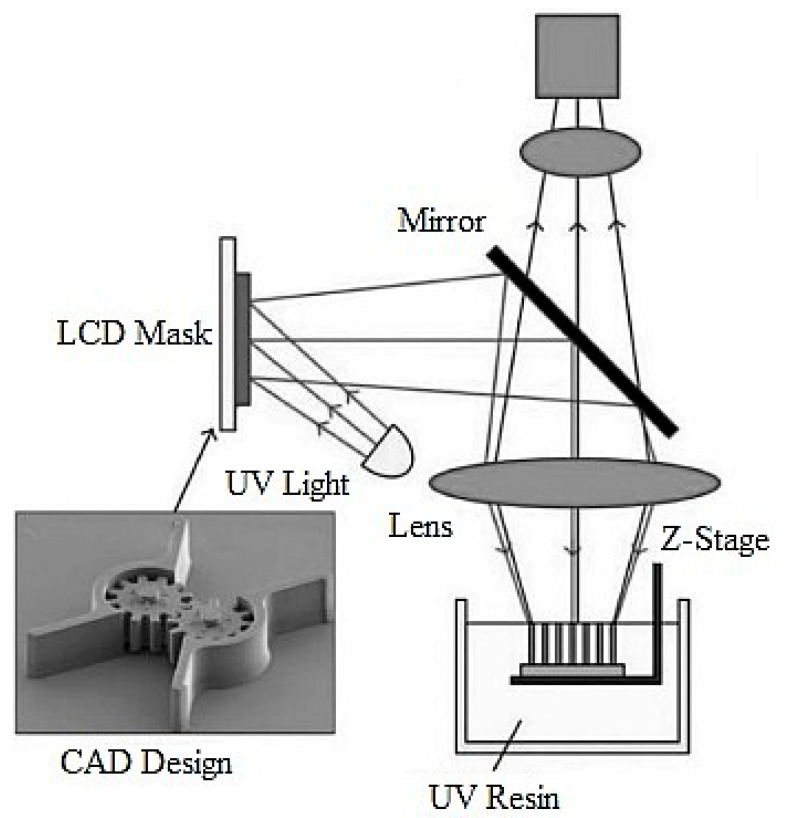
Schematic of the projection micro-stereolithography (PμSL) method [62].

Projection micro-stereolithography (PμSL) is a sophisticated 3D printing method because of its low cost, precision, velocity, and also the variety of the materials such as ceramics, biomaterials, curable photopolymer, polymer, and nanoparticle composites [54]. This method has demonstrated potential in different implementations for example micro-resonators, micro-grippers, micro-optics, biomedical micro devices, micro-fluidics, and so on [57,58,59]. Studies on PµSL are ongoing in terms of the quality and accuracy of the construction process, which affects the production of complex 3D microstructures and makes it attractive enough to be considered for commercial applications [60]. This technology begins by creating a 3D construction via a computer-assisted design program and then transforms the construction into a set of digital mask images. The working basis of PμSL is shown in Figure 3 [61]. 

Using a digital micro mirror device as the dynamic mask eliminates the cost of manufacturing a mask for each layer. Besides, the PµSL method reduces production time since each layer is produced in one exposure, and the time for mask alignment is eliminated. Moreover, this method has the least mechanical moving parts and requires only one accurate *z*-axis motorized linear stage. Consequently, PµSL decreases the cost of construction and protection [62].

All images symbolize a thin layer of the 3D structure. Along a production period, a single image is demonstrated on the reflective LCD panel. The image from the LCD is then mirrored on the liquid surface. All layers (ranging between 5–40 μm thick) are polymerized. When the layer has been solidified, it is dipped in the resin to allow a new thin layer of liquid to form. Repeating the loop forms a 3D microstructure from a layer stack. For the PμSL method, IP-S resin, which is a photopolymer, was used as the material. The properties of the photopolymer resin, which were specially developed for this 3D printer and utilized in the fabrication of the micro beam, are shown in Table 2 [54].

## 3. Fabrication

### 3.1. Fabrication with the DLP Method

**Figure 4:**
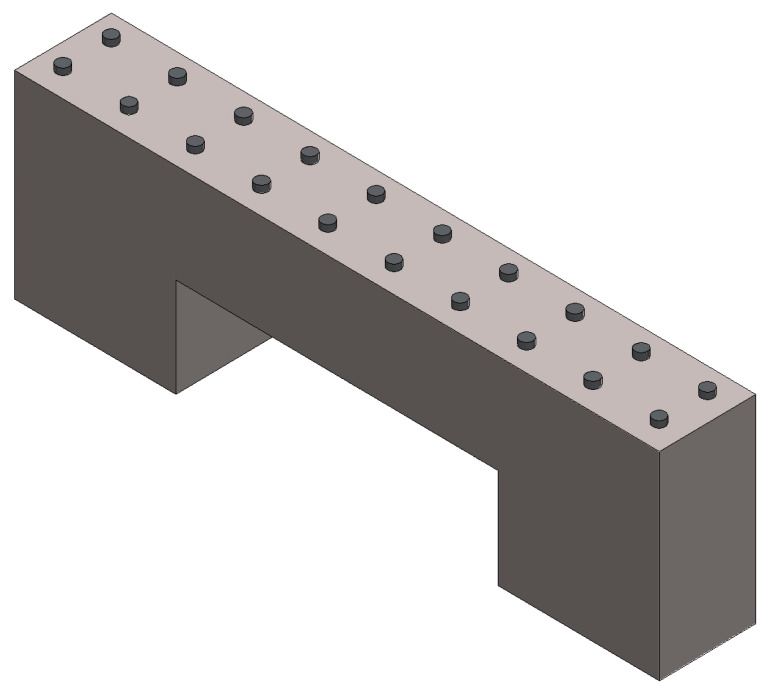
CAD design of the micro beam. The structures at the top of the design were designed as support.

**Figure 5:**
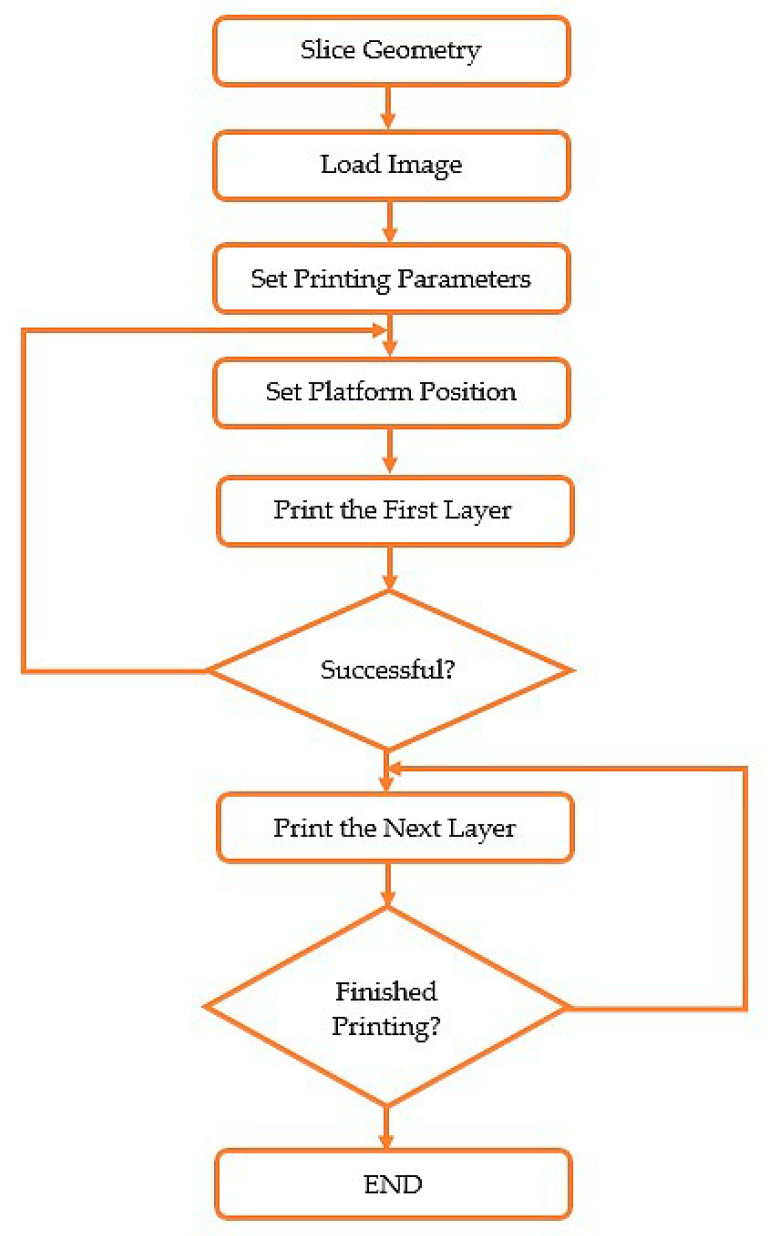
The step-by-step diagram of the 3D printing operation.

The micro beam shown in Figure 4 was fabricated by a DLP technology-based MiiCraft 3D printing device. 3D printing technology entails an input CAD model of the parts that may be designed in software or obtained from reverse engineering such as 3D scanners. When the CAD model of the micro beam is completed, it is transformed into standard STL format, which is most commonly used to represent 3D CAD models in 3D printing. In an STL file, the CAD model is symbolized using triangular facets, which are described by the x-, y-, and z-coordinates of the three vertices. The step-by-step schema of the 3D printing operation is displayed in Figure 5. The slicer first divides the object into a stack of flat layers, followed by describing these layers as linear movements of the 3D printer extruder, fixation laser, or equivalent. All these movements, together with some specific printer commands like the ones to control the extruder temperature or bed temperature, are finally written in the g-code file, that can be transferred after to the printer.

During fabrication of the micro beam, the printing parameters for example the layer thickness (LT), the light intensity (LI), and the curing time (CT) of all layer significantly affect the print quality. In this study, each parameter is selected for the printing material and TL = 30 µm, and CT = 3 s are set. When the first layer is printed, LI is set to 50% of the brightness to provide a layer bond to the platform.

**Figure 6:**
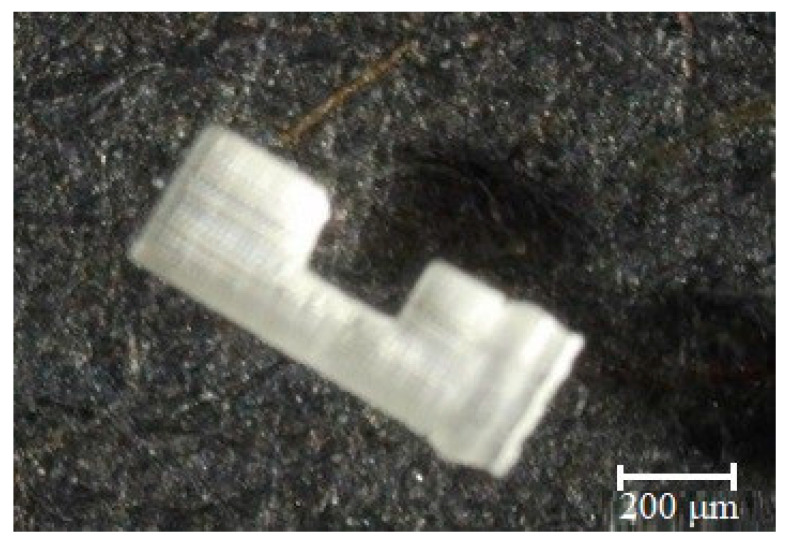
Image of the micro beam fabricated with the DLP method.

There are supports to the arms of this design. The number of supports to the arm is 20, the diameter is 3 μm, and the height is 5 μm. Some unsuccessful experiments were done before this design. Breakages were experienced during the manufacturing when the number of supports to the arm was low. Concerning the experiments, the average distance between the supports should be 10 μm to avoid breakages. When the supports are not printed correctly, they cause a collapse and break off the micro beam. An image of the micro beam manufactured with the DLP is shown in Figure 6. This image was taken with the microscope of the 3D printer device.

### 3.2. Fabrication with the PμSL Method

**Figure 7:**
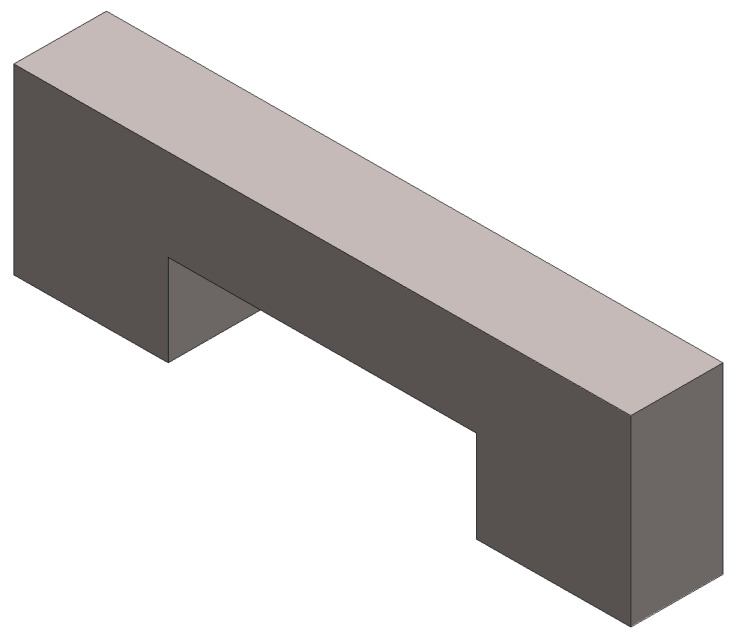
CAD design of the micro beam. The support structures under the micro beam are removed.

The micro beam shown in Figure 7 was manufactured by a projection micro-stereolithography (PμSL) method based MiiCraft 3D printing device. The manufacturing of the supported design with the PμSL method was not possible for two reasons. First, the supported structures represent the system as a 3D design. However, it is not possible to fabricate 3D structures with devices of MiiCraft 3D printing, based on PμSL technology. Second, these devices have a resolution of 65 μm and can fabricate a minimum thickness of up to 30 μm.

**Figure 8:**
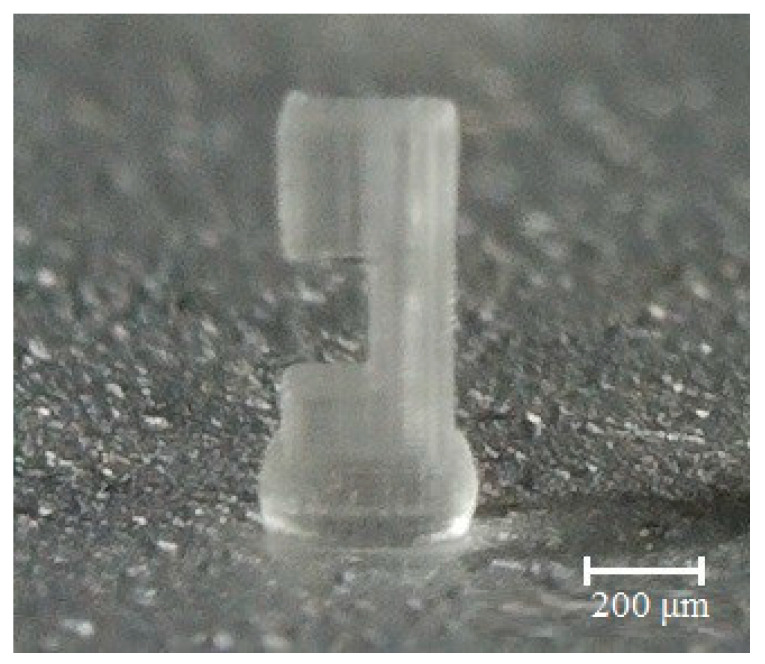
Image of the micro beam fabricated with the PμSL method.

When the support structures are removed, as shown in Figure 7, it is possible to perform the fabrication as the micro beam design can be introduced to the device (a PμSL technology-MiiCraft 3D printer) in two dimensions. An image of the micro beam manufactured with the PμSL is shown in Figure 8. This image was taken with the microscope of the 3D printer device.

## 4. Conclusions

In this study, a micro beam fabricated with conventional MEMS methods, was manufactured for the first time using the DLP and PμSL methods, which are 3D printing procedures. First, the printable scale of the DLP 3D printing method was evaluated, and it demonstrated that the printer could produce structures with a size of 86.7 μm.

The experimental studies showed that 3 μm diameter supports were fabricated with the DLP method. However, they could not be fabricated with the PμSL method even when the diameters of the supports were 3 μm. After these support structures were removed, the micro beam was fabricated with PμSL. It was determined that PμSL was not suitable for complex structures. The results show the success of the 3D printer and the suitability of manufacturing a micro beam using the DLP printing method with fast and high sensitivity.

As a result of this study, it was found that DLP is more appropriate because it allows the manufacturing of complex 3D structures with smaller dimensions, while PμSL is only suitable for simple 2D microstructures. It is expected that this paper will contribute to the literature in terms of fabrication of a micro device through the use and comparison of different techniques.

## Figures and Tables

**Table 1:** Description of the micro beam.

Parameter	Symbol	Value (μm)
Length of the feet	$L_{f}$	25
Length of the arm	$L_{a}$	50
Height of the arm	$w_{a}$	25
Height of the feet	$w_{f}$	50
Thickness of the beam	t	30

**Table 2:** The elemental composition and fundamental features of the IP-S resin [54].

**Chemical Properties of Resin**				
**Carbon (at%)**	**Hydrogen (at%)**	**Nitrogen (at%)**	**Oxygen (at%)**	**Empirical Formula**
31.45	54.07	5.75	11.7	CH_1.71_N_0.085_O_0.35_
**Physical and Mechanical Features**				
**Density (liq) g/(cm^3^)**	**Density (s) g/(cm^3^)**	**Young’s Modulus (GPa)**	**Hardness (MPa)**	**Refractive Index**
1.3	1.5	3.8	150	1.51
